# *Rickettsia* association with two *Macrolophus* (Heteroptera: Miridae) species: A comparative study of phylogenies and within-host localization patterns

**DOI:** 10.3389/fmicb.2022.1107153

**Published:** 2023-02-23

**Authors:** Maria Dally, Yehuda Izraeli, Eduard Belausov, Netta Mozes-Daube, Moshe Coll, Einat Zchori-Fein

**Affiliations:** ^1^Department of Entomology, RH Smith Faculty of Agriculture, Food and Environment, Hebrew University of Jerusalem, Rehovot, Israel; ^2^Department of Entomology, Newe-Ya’ar Research Center, ARO, Ramat-Yishay, Israel; ^3^Department of Evolution and Environmental Biology, University of Haifa, Haifa, Israel; ^4^The Institute of Plant Sciences, The Volcani Center, ARO, Rishon LeZion, Israel

**Keywords:** FISH, *Macrolophus melanotoma*, *Macrolophus pygmaeus*, omnivory, *Rickettsia bellii*, *Rickettsia limoniae*

## Abstract

Many arthropods host bacterial symbionts, some of which are known to influence host nutrition and diet breadth. Omnivorous bugs of the genus *Macrolophus* (Heteroptera: Miridae) are mainly predatory, but may also feed on plants. The species *M. pygma*eus and *M. melanotoma* (=*M. caliginosus*) are key natural enemies of various economically important agricultural pests, and are known to harbor two *Rickettsia* species, *R. bellii* and *R. limoniae*. To test for possible involvement of symbiotic bacteria in the nutritional ecology of these biocontrol agents, the abundance, phylogeny, and distribution patterns of the two *Rickettsia* species in *M. pygmaeus* and *M. melanotoma* were studied. Both of the *Rickettsia* species were found in 100 and 84% of all tested individuals of *M. pygmaeus* and *M. melanotoma,* respectively. Phylogenetic analysis showed that a co-evolutionary process between *Macrolophus* species and their *Rickettsia* is infrequent. Localization of *R. bellii* and *R. limoniae* has been detected in both female and male of *M. pygmaeus* and *M. melanotoma*. FISH analysis of female gonads revealed the presence of both *Rickettsia* species in the germarium of both bug species. Each of the two *Rickettsia* species displayed a unique distribution pattern along the digestive system of the bugs, mostly occupying separate epithelial cells, unknown caeca-like organs, the Malpighian tubules and the salivary glands. This pattern differed between the two *Macrolophus* species: in *M. pygmaeus, R. limoniae* was distributed more broadly along the host digestive system and *R. bellii* was located primarily in the foregut and midgut. In contrast, in *M. melanotoma*, *R. bellii* was more broadly distributed along the digestive system than the clustered *R. limoniae*. Taken together, these results suggest that *Rickettsia* may have a role in the nutritional ecology of their plant-and prey-consuming hosts.

## 1. Introduction

Insects are known to form successful long-term symbioses with endosymbiotic bacteria (Harris et al., 2010; Gupta and Nair, 2020). Such bacteria may be referred to as primary symbionts, if they are obligatory and thus essential for the survival of the host (Sudakaran et al., 2017), or as secondary symbionts, if they are not involved in functions essential for host survival or reproduction. Facultative association with secondary symbionts may affect host biology and ecology by influencing host fitness through altered traits and capabilities (Sudakaran et al., 2017).

*Rickettsia* (Alphaproteobacteria: Rickettsiales) are gram-negative obligate intracellular bacteria found within eukaryotic cells; they have a variety of interactions with their arthropod hosts (Weinert et al., 2015; El Karkouri et al., 2022). *Rickettsia* can be found in many insect tissues, including Malpighian tubules, gut compartments, oocytes, and, rarely, sperm cells (Machtelinckx et al., 2012; Watanabe et al., 2014; Dally et al., 2020). While *Rickettsia* were initially recognized as human and animal pathogens transmitted by blood-feeding arthropods such as ticks, mites, lice and fleas, in recent decades most species and strains have been shown to be non-pathogenic endosymbionts of arthropods (Weinert et al., 2015; Pilgrim et al., 2021).

Most insect hosts of *Rickettsia* belong to the orders Hemiptera, Coleoptera, Diptera and Hymenoptera (Weinert et al., 2015), in all of which the bacteria have a positive effect on host survival and reproductive success. In the pea aphid *Acyrthosiphon pisum* (Hemiptera: Aphididae), for instance, *Rickettsia* improve host resistance to a pathogenic *Pandora* fungus (Lukasik et al., 2013). In the sweet potato whitefly *Bemisia tabaci* (Hemiptera: Aleyrodidae), *Rickettsia* provide protection against *Pseudomonas syringae* infection (Hendry et al., 2014), and enhance fertility, longevity and development (Himler et al., 2011). Additionally, these bacteria stimulate oogenesis in the booklouse *Liposcelis bostrychophila* (Psocoptera: Liposcelididae) (Perotti et al., 2006). *Rickettsia* may also have negative effects on their hosts, for example by slowing development (Semiatizki et al., 2020) or inducing male-killing in the host (Werren et al., 1994; Giorgini et al., 2010).

Within the suborder Heteroptera (Order Hemiptera), the family Miridae includes some 10,000 species with a wide range of feeding habits including herbivory, carnivory, and omnivory (Wheeler, 2002). Several members of this family, such as *Nesidiocoris tenuis, Macrolophus pygmaeus* and *M. melanotoma*, serve as biological control agents against key crop pests (Schaefer and Panizzi, 2000; Castañé et al., 2011), yet these mainly predaceous species may also feed on plant materials (Perdikis and Lykouressis, 2000). The potential of these predators to reduce agricultural yields when prey is scarce has limited their use in biological control programs (Castañé et al., 2011; Moerkens et al., 2016). It is therefore important to explore the presence and role of symbionts in the nutritional ecology of these omnivorous biological control agents.

Omnivores, like most insects, serve as hosts to symbiotic bacteria. In previous studies, *M. pygmaeus* was found to harbor *Wolbachia* and two species of *Rickettsia*, *R. bellii and R. limoniae.* In contrast, *M. melanotoma* was observed to house only *Wolbachia* and *R. limoniae.* Regarding symbiont distribution, *Wolbachia* are located in the ovaries of *M. pygmaeus*, where they induce cytoplasmic incompatibility (Machtelinckx et al., 2009, 2012). The two *Rickettsia* species, in contrast, were found in our previous study to be distributed in both the digestive and reproductive systems of *M. pygmaeus*, each displaying a unique cellular occupancy and a specific distribution pattern along the digestive system compartment (Dally et al., 2020). The significance of these differences in distribution is not yet clear, but a link to the omnivorous host diet has been proposed. The current study revealed the presence of both *Rickettsia* species, *R. bellii* and *R. limoniae*, in *M. melanotoma*, in contrast with the earlier findings of Machtelinckx et al. (2012). Accordingly, our objective is to further explore the role of *Rickettsia* in omnivore diet and nutrition. To this end, the occurrence of *Rickettsia* species in *M. melanotoma* was described, their localization patterns were compared to those found in *M. pygmaeus*, and *Rickettsia* abundance and phylogeny in the two hosts were determined.

## 2. Materials and methods

### 2.1. Insect origin, DNA extraction, and verification of insect identity

For this study, 82 females of *M. melanotoma* were collected from *Dittrichia viscosa* (Asteraceae) in various locations in northern and south-central Israel (see Supplementary additional file 1, table of collection sites of *M. melanotoma* females in Israel). The collected insects were placed immediately in 100% ethanol, and then stored at-20°Cuntil analysis. A culture of *M. pygmaeus* was established in February 2018 with 30 adult females and 20 adult males obtained from a commercial biological control company (BioBee Sde Eliyahu Ltd., Israel), with occasional infusion of additional insects from the same source (for details see Dally et al., 2020). DNA was extracted from individual insects using the Nucleospin Tissue XS Kit (“Macherey-Nagel,” Switzerland), following the manufacturer’s instructions.

The identity of field-collected *M. melanotoma* males and females was determined under a stereoscopic microscope, based on morphological characters presented by Martinez-Cascales et al. (2006). In addition, the mitochondrial cytochrome oxidase I (COI) gene fragment was used to verify species identity. COI was amplified from all individuals and sequenced individually using LCO1490 and HCO2198 primers (Table 1). Sterilized water and DNA of *Bemisia tabaci* served as negative and positive controls, respectively. PCR procedures were carried out following the protocol described by Dally et al. (2020), and sequencing was performed using an automatic sequencer (ABI 3700 DNA analyser, Macrogen Inc.). The resulting sequences were compared with known sequences in the databases, using BLAST searches, and deposited in NCBI GenBank, under accession numbers OQ374915-OQ374918, OQ374921-OQ374924, OQ398710, OQ398711,OQ271380-OQ271381 and OQ410975.

**Table 1:** Primer and probe sequences used in this study for PCR analyses and fluorescence *in situ* hybridization of *Rickettsia* endosymbionts.

Gene	*Rickettsia* species	Name	Sequence	Tm	Reference
*16SrRNA*	*Rickettsia bellii*	Rb-F	5’-GCTCAGAACGAACGCTATC-3’	58°C	Gottlieb et al. (2006)
		Rb-R	5’-GAAGGAAAGCATCTCTGC-3’		
		Belli-F1	5’-AGAAAAAGCCCCGGCTAACTCC-3’		This study
		Belli-F2	5’-TTACTTGCAGAAAAAGCCCC-3’		This study
		1,044-R	5′- TTTTCTTATAGTTCCTGGCATTACCC-3’		Caspi-Fluger et al. (2012)
	*Rickettsia limoniae*	*Rick limoniaeF*	5′- CGGTACCTGACCAAGAAAGC-3′	55°C	Machtelinckx et al. (2012)
		Riclim416R	5’-GCTTTCTTGGTCAGGTACCG-3’		This study
	All	F27	5’-AGAGTTTGATCMTGGCTCAG-3’	57°C	Weisburg et al. (1991)
		1491R	5’-CTACGGCTACCTTGTTACGA-3′		
*GltA*	*R. bellii*	GltA133F	5′- GGTTTTATGTCTACTGCTTCKTG-3’	54°C	Machtelinckx et al. (2012)
		GltA1197R	5′- CATTTCTTTCCATTGTGCCATC-3′		
	*R. limoniae*	GltAlimF	5’-GTAGAAGAAAATGAACG-3’	55°C	This study
		GltA1193R	5’-TCTTTCCATTGCCCC-3’		Pilgrim et al. (2021)
*CoxA*	*R. bellii*	CoxA322F	5′- GGTGCTCCTGATATGGCATT-3′	54°C	Machtelinckx et al. (2012)
		CoxA1413R	5′- CATATTCCAACCGGCAAAAG-3′		
	*R. limoniae*	CoxA39F	5’-CGGCTTTTGTTGATGGTGGTG-3’	55°C	This study
		CoxA233F	5’-CGATGGTATGGGGTTATTTG-3’		
		CoxA900R	5’-GCCCATCATTTCAGGATATTGTC-3’		
*COI*	All	LCO1490	5’-GGTCAACAAATCATAAAGATATTGG-3’	55°C	Simon et al. (1994)
		HCO2198	5’-TAAACTTCAGGGTGACCAAAAAATCA-3’		
Probes					
	*R. belli*	Rb1-Cy3	5’-TCCACGTCGCCGTCTTGC-3’		Gottlieb et al. (2006)
	*R. limoniae*	Rl1-Cy5	5′- GCTTTCTTGGTCAGGTACCG-3’		This study

### 2.2. *Rickettsia* prevalence in *Macrolophus melanotoma*

The abundance of *R. bellii* and *R. limoniae* in *M. pygmaeus* was reported by Dally et al. (2020). PCR was used to assess the prevalence of the two *Rickettsia* species in *M*. *melanotoma.* The DNA extracted from each of the collected individuals was screened with species-specific primers for the *16S rRNA* gene of *R. bellii* and *R. limoniae* (as describe above; Table 1). DNA of *M. pygmaeus* harboring the bacteria served as a positive control for both bacterial species, following Dally et al. (2020).

### 2.3. Phylogenetic analysis

#### 2.3.1. Phylogenetic analysis of *Rickettsia*

The phylogenetic relationships between the two *Rickettsia* species were inferred from concatenated sequences of a minimum of two of the following three genes: *16S rRNA*, *GltA* (citrate synthase) and *CoxA* (cytochrome oxidase c subunit 1) (primer pairs are detailed in Table 1). Consensus sequences were obtained using DNAman software and were deposited in NCBI GenBank, under accession numbers OQ374915-OQ374918, OQ374921-OQ374924, OQ398710, OQ398711,OQ271380-OQ271381 and OQ410975. Representative sequences were chosen from Pilgrim et al. (2021) (see Supplementary additional files 2, 3; accession numbers used for *Rickettsia* phylogenetic analyses). The total length of concatenated sequences was in the range of ~2,100–3,000 bp. Multiple sequence alignment was conducted by MAFFT using default parameters, and a maximum likelihood tree was constructed with the GTR substitution model using PhyML, with branch support measured by approximate likelihood ratio tests (SH-aLRT; (Guindon et al., 2010). *Rickettsia japonica* (accession number AP017600.1) was used as an outgroup for both *R. limoniae* and *R. bellii* trees.

#### 2.3.2. Phylogenetic analysis of *Macrolophus* spp.

The phylogenetic relationships between the two studied *Macrolophus* species and other members of the Miridae were investigated using the mitochondrial cytochrome oxidase subunit I (COI) gene. A ~ 650 bp-long fragment was sequenced using the primers LCO1490, HCO2198 (Table 1). Representative sequences were chosen from a BLASTN search targeted to Miridae (taxid no. 30083) (See Supplementary additional file 4; accession numbers used for Mitochondrial *COI* phylogenetic analyses). Multiple sequence alignment was conducted by MAFFT using default parameters, and a maximum likelihood tree was constructed with the GTR substitution model using PhyML, with 100 replicates for bootstrap support. *Orius laevigatus* (Heteroptera: Anthocoridae) was used as an outgroup.

### 2.4. Morphology of the gut, ovaries, and salivary glands

To characterize the morphology of various relevant *M*. *melanotoma* organs, more than 40 adult females and 40 adult males were dissected under a stereomicroscope. Dissections were photographed with a 3-D digital microscope (see description in Dally et al., 2020).

### 2.5. Localization of *Rickettsia bellii* and *Rickettsia limoniae* in *Macrolophus melanotoma*

Fluorescent *in situ* hybridization (FISH) was performed to determine the location of *R. limoniae* and *R. bellii* in the reproductive organs, digestive tract and salivary glands. The protocol described by Gottlieb et al. (2006) was followed with slight modifications for 50 mounted samples (see Dally et al., 2020). Images were acquired using an OLYMPUS IX 81 (Japan) inverted laser scanning confocal microscope (FLUOVIEW 500) equipped with 405, 561, 640 nm laser lines, a UplanApo 10 x/0.4 NA dry objective, and PlanApo 40 x/0.9 NA and 60 ×/1.0 NA water immersion objectives (see description in Dally et al., 2020). Confocal optical sections were obtained at increments of 5 μm, 2.5 μm, 1.3 μm and 0.8 μm for 10x, 20x, 40x and 60x objectives, respectively.

## 3. Results

### 3.1. Species verification

BLAST searches to all consensus sequences of the COI gene of the field-collected *M*. *melanotoma* and laboratory *M. pygmaeus* exhibited over 99.5% sequence similarity to available sequences of the matching *Macrolophus* species, thus verifying species identification.

### 3.2. *Rickettsia* prevalence in *Macrolophus melanotoma*

Out of the 82 *M. melanotoma* adults screened by diagnostic PCR, 84% (*n* = 69) were found to be positive for both *Rickettsia* species. *R. bellii* was detected alone in 10% (*n* = 8) of the samples, *R. limoniae* alone was detected in 1% (*n* = 1), and no *Rickettsia* were found in 5% (*n* = 4) of the adults. As previously reported, in *M. pygmaeus* both *Rickettsia* species were present in 100% of the tested adults (Dally et al., 2020).

### 3.3. Phylogenetic analysis

The aim of the analysis was to examine the overall phylogenetic assignment of the studied *Macrolophus* strains and *Rickettsia* symbionts, and to assess whether any co-evolutionary pattern could be identified between the symbionts and their corresponding hosts.

#### 3.3.1. *Macrolophus* species

**Figure 1:**
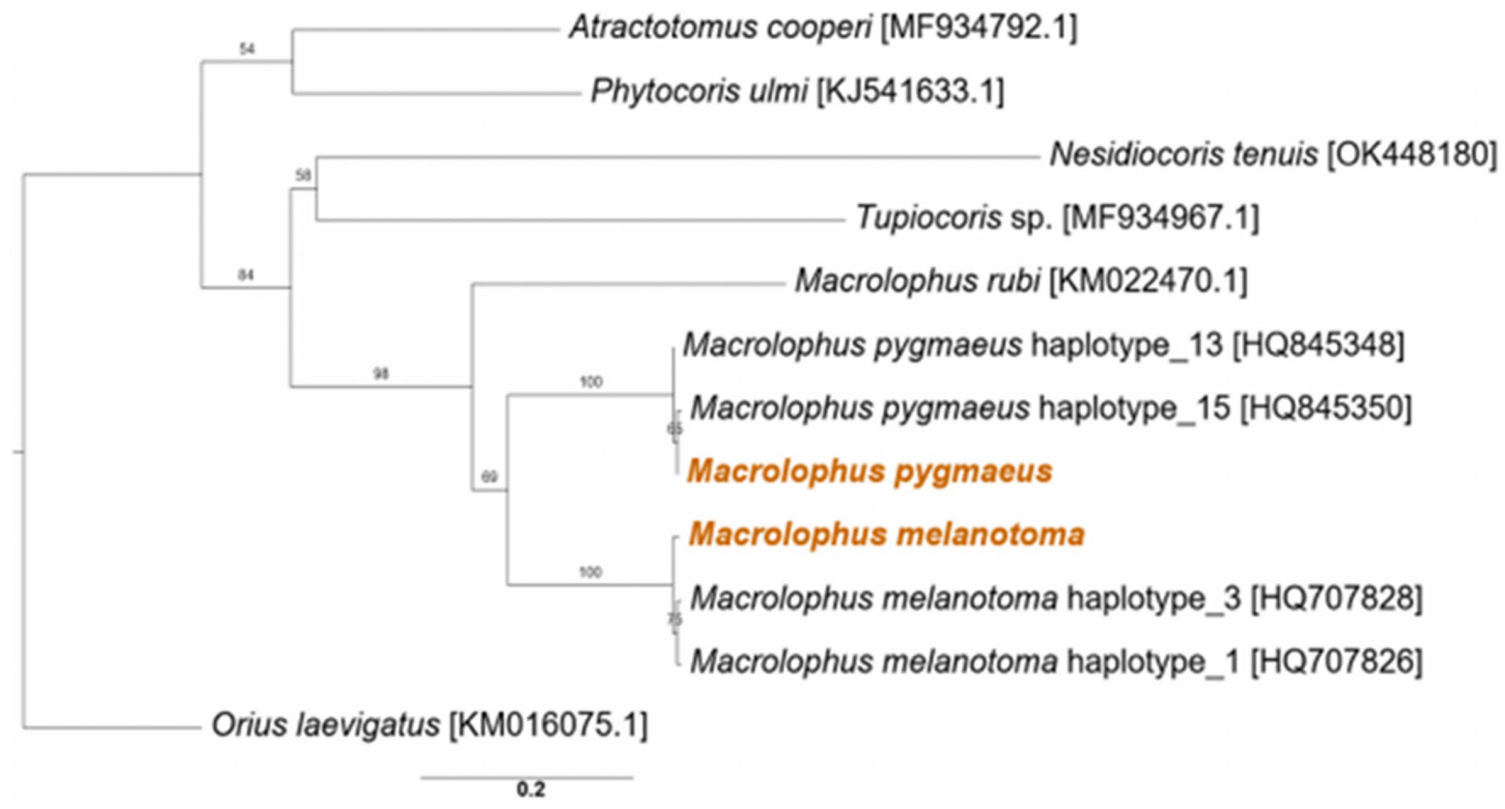
Maximum likelihood phylogeny of *Macrolophus* spp. from the present study (colored) and other species in the Miridae family, based on the COI gene. Accession numbers of the reference sequences are in brackets. The outgroup is *Orius laevigatus* (Hemiptera: Anthocoridae). Bootstrap support values of 100 repeats are indicated. Scale bar indicates 0.2 substitutions per site.

Analysis of the phylogenetic relationships between the two studied *Macrolophus* species and other members of the Miridae confirmed the BLAST search results and morphological identification; each of them clustered together with other haplotypes of the relevant species, and the overall tree was fitted to the taxonomic assignments of other Miridae members (Figure 1).

#### 3.3.2. *Rickettsia* species

**Figure 2:**
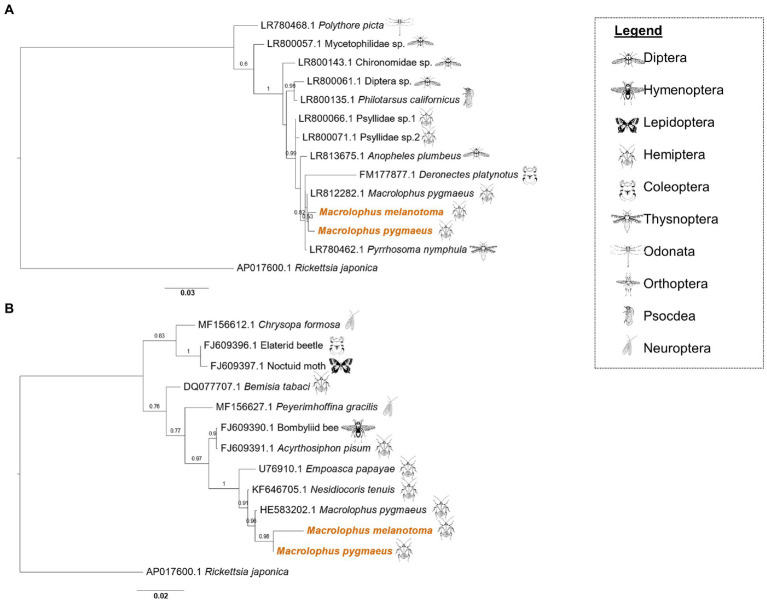
Maximum likelihood phylogeny of *Rickettsia limoniae* and *Rickettsia bellii* from *Macrolophus melanotoma* and *Macrolophus pygmaeus*. **(A)** Phylogeny of *R. limoniae* in the present study (colored) and other insect species, inferred from concatenated sequences of *16S rRNA*, *GltA,* and *CoxA* genes. Scale bar = 0.03 substitutions per site. **(B)** Phylogeny of *R. bellii*. The analysis of all *R. bellii* strains was inferred from concatenated sequences of *16S rRNA*, *GltA* and *CoxA* genes, except that the concatenate of *M. melanotoma* lacks the *16S rRNA* sequence. Names at the tips indicate the insect host of the specific strain, and numbers indicate the accession number of the *16S rRNA* gene. Accession numbers of the two other genes included in the concatenate can be found in Supplementary files 2 and 3. Scale bar = 0.02 substitutions per site. Insect icons indicate the insect order of the host.

The three genes from each of the *Rickettsia* species sequenced from *M. melanotoma* specimens collected in all five localities in Israel, had over 99% similarity between them, indicating that *M. melanotoma* from all locations is infected with the same strains of *R. bellii* and the same strains of *R. limoniae*. Accordingly, we constructed consensus sequences for each of the *Rickettsia* strains from all field collected specimens of *Macrolophus*. Concatenate sequences of three *R. limoniae* and *R. bellii* genes from each *Macrolophus* species were constructed to assess whether any co-evolutionary pattern could be identified between the symbionts and their corresponding hosts. *R. limoniae* inhabiting *M. pygmaeus* and *M. melanotoma* showed greater similarity to each other than to any of the other chosen reference sequences (Figure 2A). Pairwise alignment between them was 96%, while alignment with all other sequences ranged from 70 to 92% (alignment with the outgroup was 54%). The studied strains clustered together with an *R. limoniae* strain from *Deronectes platynotus*, a dytiscid beetle. Strains from other heteropterans, including a strain from *M. pygmaeus* obtained by Pilgrim et al. (2021), were distributed along the branches of the tree, without a pattern resembling hosts’ phylogeny.

As in *R. limoniae*, *R. bellii* clustered together on the phylogenetic tree (Figure 2B). Pairwise alignment of the *M. pygmaeus* strain to others on the tree ranged from 62% (*Bemisia tabaci* [DQ077707.1]) to 98% (*M. pygmaeus* from another study [HE583203.1]). Interestingly, while the *CoxA* and *GltA* sequences of the *R. bellii* symbiont in *M. melanotoma* were similar to other sequences from the bellii group, the *16S rRNA* gene was more similar to sequences obtained from the limoniae group (Supplementary Figure 1).

### 3.4. Morphology of the gut, ovary, and salivary gland

**Figure 3:**
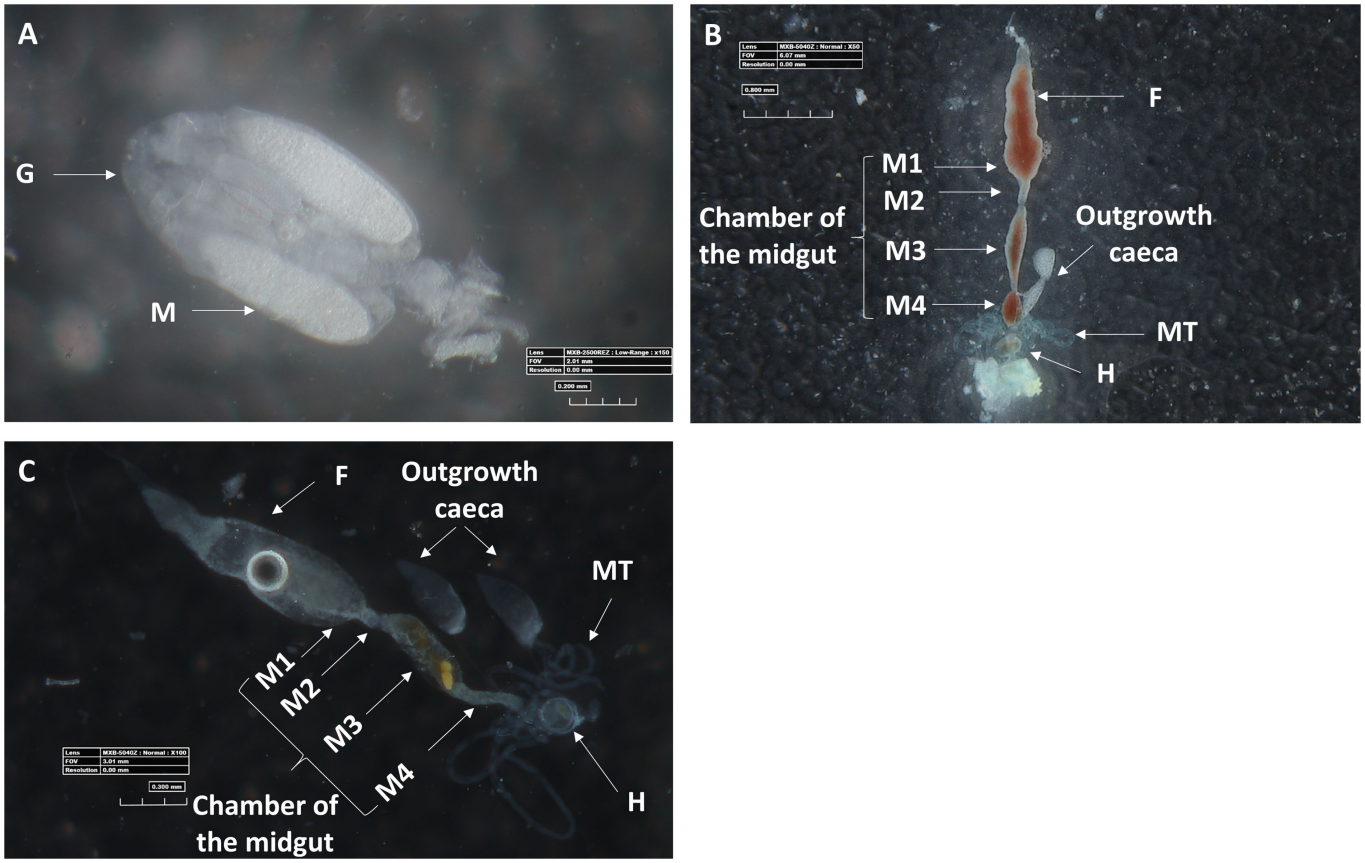
Three-D digital microscopic images of *M. melanotoma* telotrophic ovary, and female and male digestive systems. **(A)** An isolated ovary with several ovarioles; M, mature oocyte; G, Germarium, **(B)** Isolated female and **(C)** male digestive systems; F, foregut; M1, midgut first region; M2, midgut second region; M3, midgut third region; M4, midgut fourth region (with outgrowth caeca); MT, Malpighian tubules; H, hindgut.

Microscopic observation of the ovaries and digestive system of *M. melanotoma* revealed similarity to those previously described in *M. pygmaeus* (Figures 3A–C; Dally et al., 2020). The digestive tract consists of a tubular foregut with a direct opening to the mouth; a large sac-like anterior part of the midgut; a second tubular region of the midgut; a soft, somewhat swollen third midgut region; and the posterior fourth midgut region, also moderately swollen, which connects to the hindgut at the point of attachment of the Malpighian tubules (Figures 3B,C). Two caeca-like organs, which appear to be larger in males than in females, are connected to the posterior end of the fourth midgut region in the area of the Malpighian tubule openings (M4 in Figures 3B,C).

**Figure 4:**
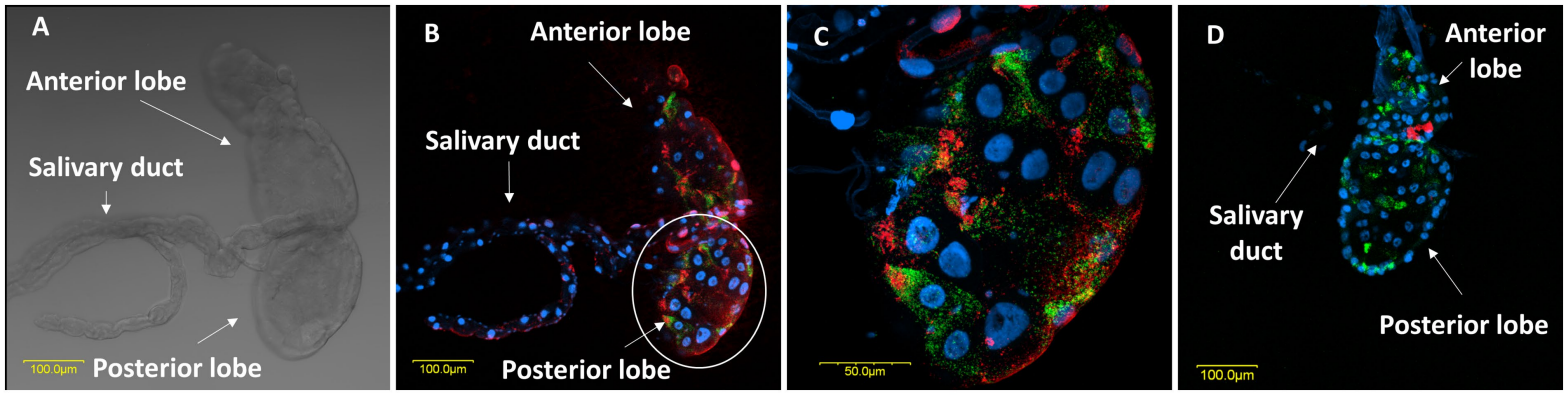
**(A)** A light transmitted microscopic image of a salivary gland in a *M. melanotoma* female, showing the anterior and posterior lobes of the salivary gland, and the salivary duct. FISH of the salivary glands of *M. melanotoma* **(B)** and *M. pygmaeus* **(D)** females. DNA in blue, *R. bellii* in red, and *R. limoniae* in green. **(C)** Enlarged region of the posterior lobe of the salivary gland of *M. melanotoma* female (white circle in B). Images **(B–D)** represent serial Z section of 15 μm, 27 μm, and 20 μm, respectively.

Microscopic observations further revealed the structural configuration of the salivary glands of female *M*. *melanotoma* and *M. pygmaeus*. Mirids, like all terrestrial heteropterans, have a pair of salivary glands located in the thorax, next to the alimentary canal (Wheeler, 2002). The system appeared to have similar morphology in both bug species, with two symmetrical salivary glands, each composed of an anterior lobe, a posterior lobe, and a salivary duct (Figure 4A).

### 3.5. Localization of *Rickettsia bellii* and *Rickettsia limoniae* in *Macrolophus pygmaeus* and *Macrolophus melanotoma*

**Figure 5:**
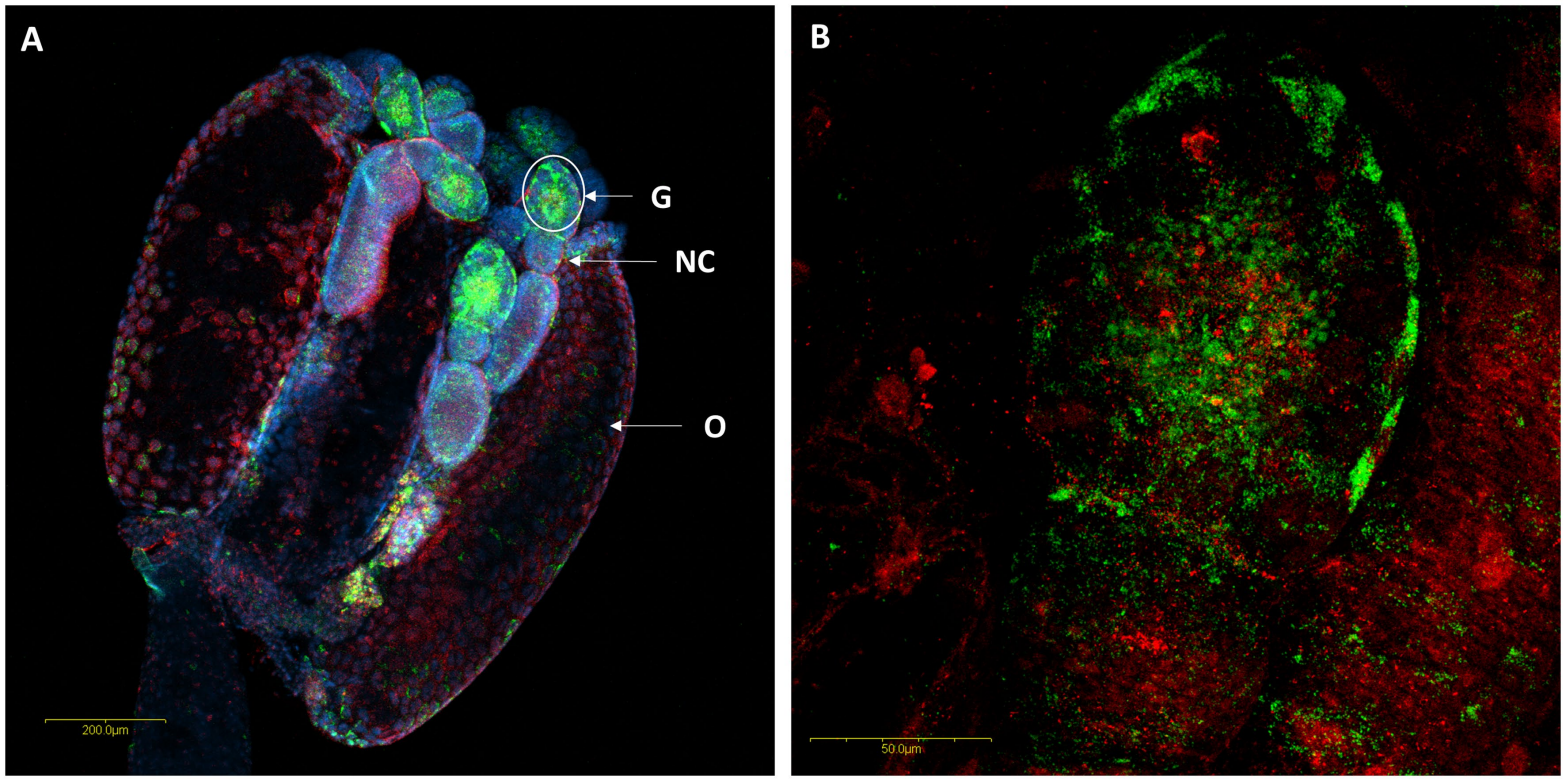
FISH of *M. melanotoma* telotrophic ovarioles with *R. bellii* specific probes (red), *R. limoniae* specific probes (green), and DNA dye (blue). **(A)** Ovary with several ovarioles, *R. bellii* and *R. limoniae* are concentrated in the germarium; G, Germarium; NC, Nurse cells; O, Oocyte. **(B)** Enlarged region of the germarium (white circle in A). Images **(A,B)** represent serial Z section of 40 μm and 26 μm, respectively.

*In situ* hybridization targeting bacterial *16S rRNA* allowed visualization of the two *Rickettsia* species within the ovaries, digestive tract and salivary gland of *M. pygmaeus and M. melanotoma*. In the ovaries, *R. bellii* and *R. limoniae* were found to be concentrated mainly in the germarium (Figure 5A) and scattered therein (Figures 5A,B).

**Figure 6:**
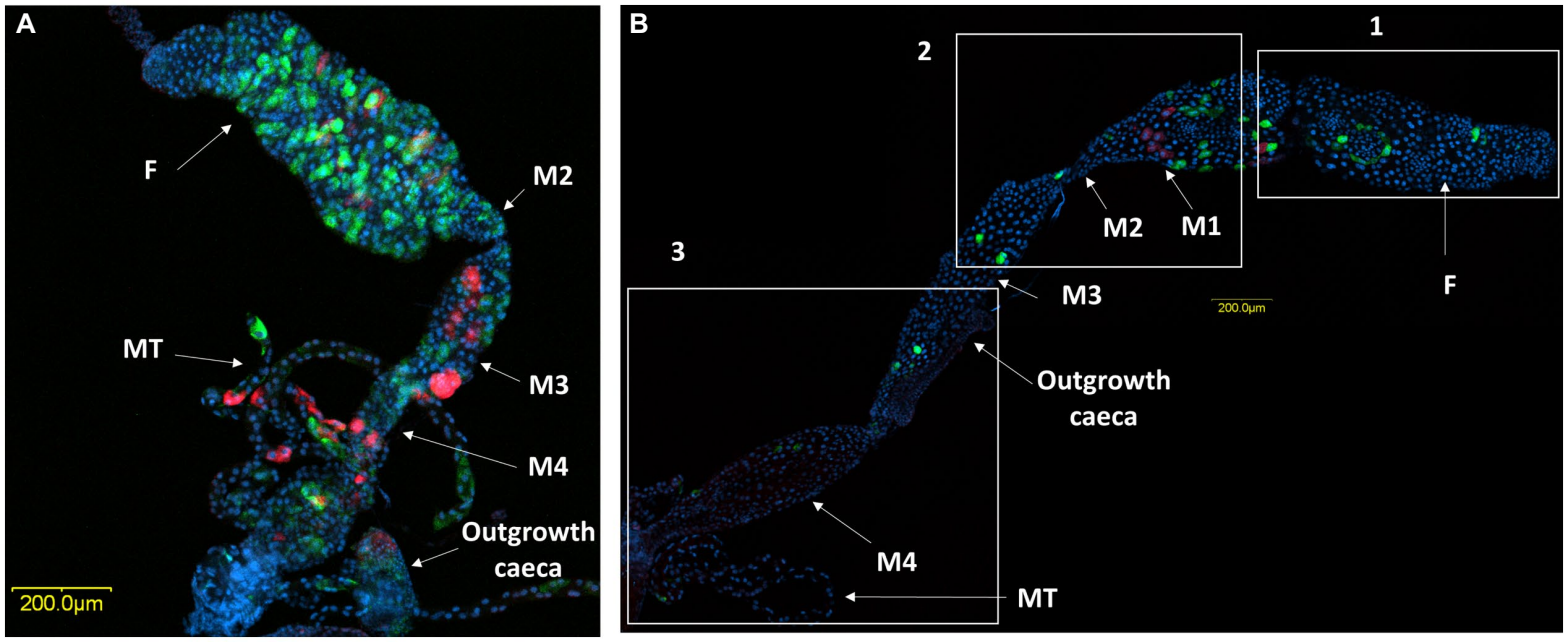
FISH of *M. pygmaeus* digestive system (DNA in blue). Male **(A)** and female **(B)** digestive systems, *R. bellii* (red), *R. limoniae* (green). Reconstruction: three frames of the same gut **(B)**; rectangle 1 picture number 1, rectangle 2 picture number 2, rectangle 3 picture number 3. Images A and B number 1, B number 2, B number 3 represent serial Z section of 45 μm, 25 μm, 55 μm and 60 μm respectively; F, foregut; M1, midgut first region; M2, midgut second region; M3, midgut third region (with outgrowth caeca); M4, midgut fourth region; MT, Malpighian tubules.

**Figure 7:**
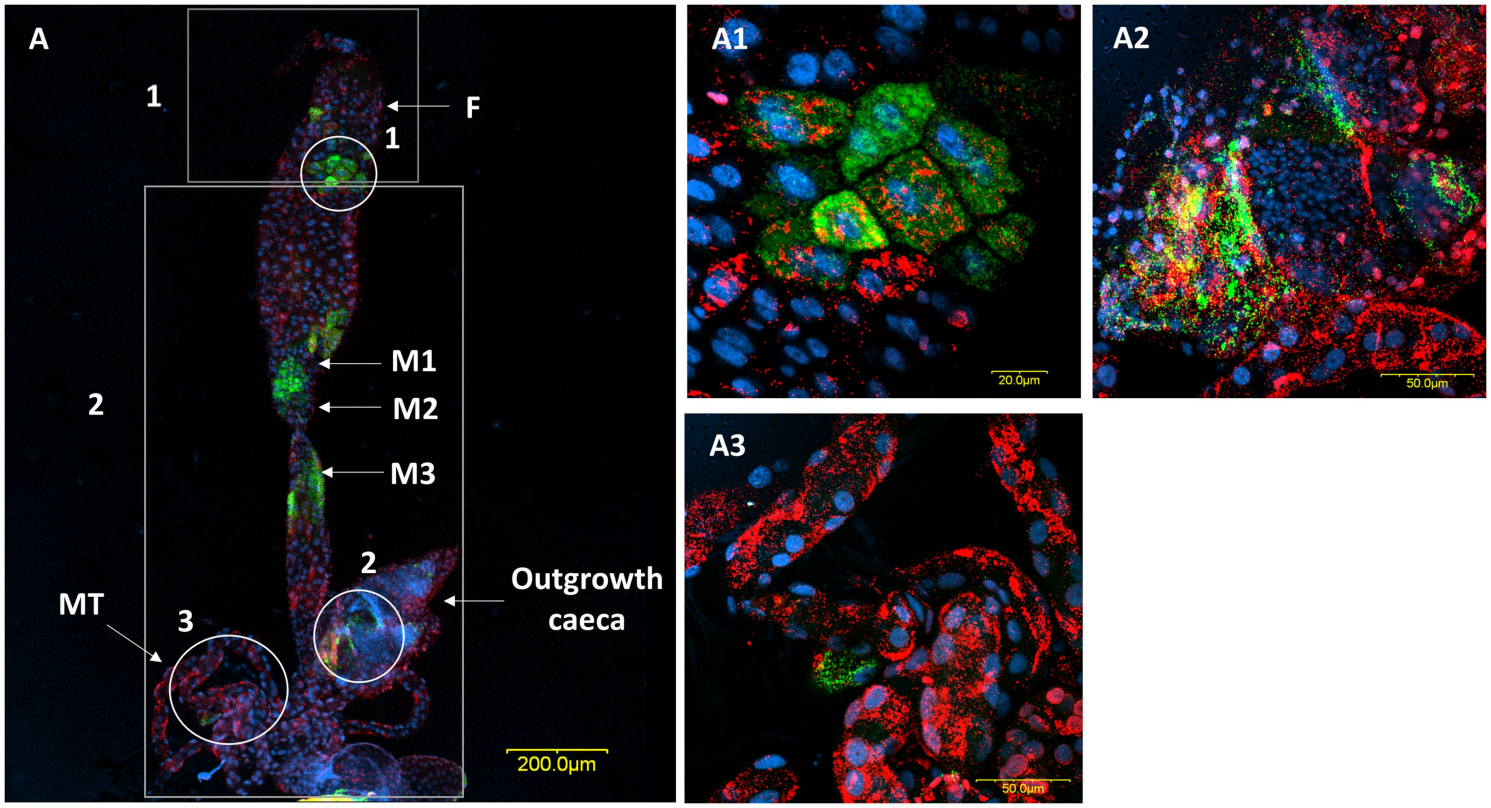
FISH of a male digestive system (DNA in blue). **(A)** The digestive system, *R. bellii* (red), *R. limoniae* (green). Reconstruction: two frames of the same gut (A); rectangle 1 picture number 1, rectangle 2 picture number 2. (**A1)** Enlarged region from the foregut (see circle 1 in A). **(A2)** Enlargement of the outgrowth caeca region, in the gut tissue of the fourth midgut region (see circle 2 in A). **(A3)** Enlarged region of Malpighian tubules (see circle 3 in A). Images A number 1, A number 2, and **(A1–A3)** represent serial Z section of 40 μm, 40 μm, 6.4 μm, 11.2 μm, 28 μm respectively; F, foregut; M1, midgut first region; M2, midgut second region; M3, midgut third region; MT, Malpighian tubules.

**Figure 8:**
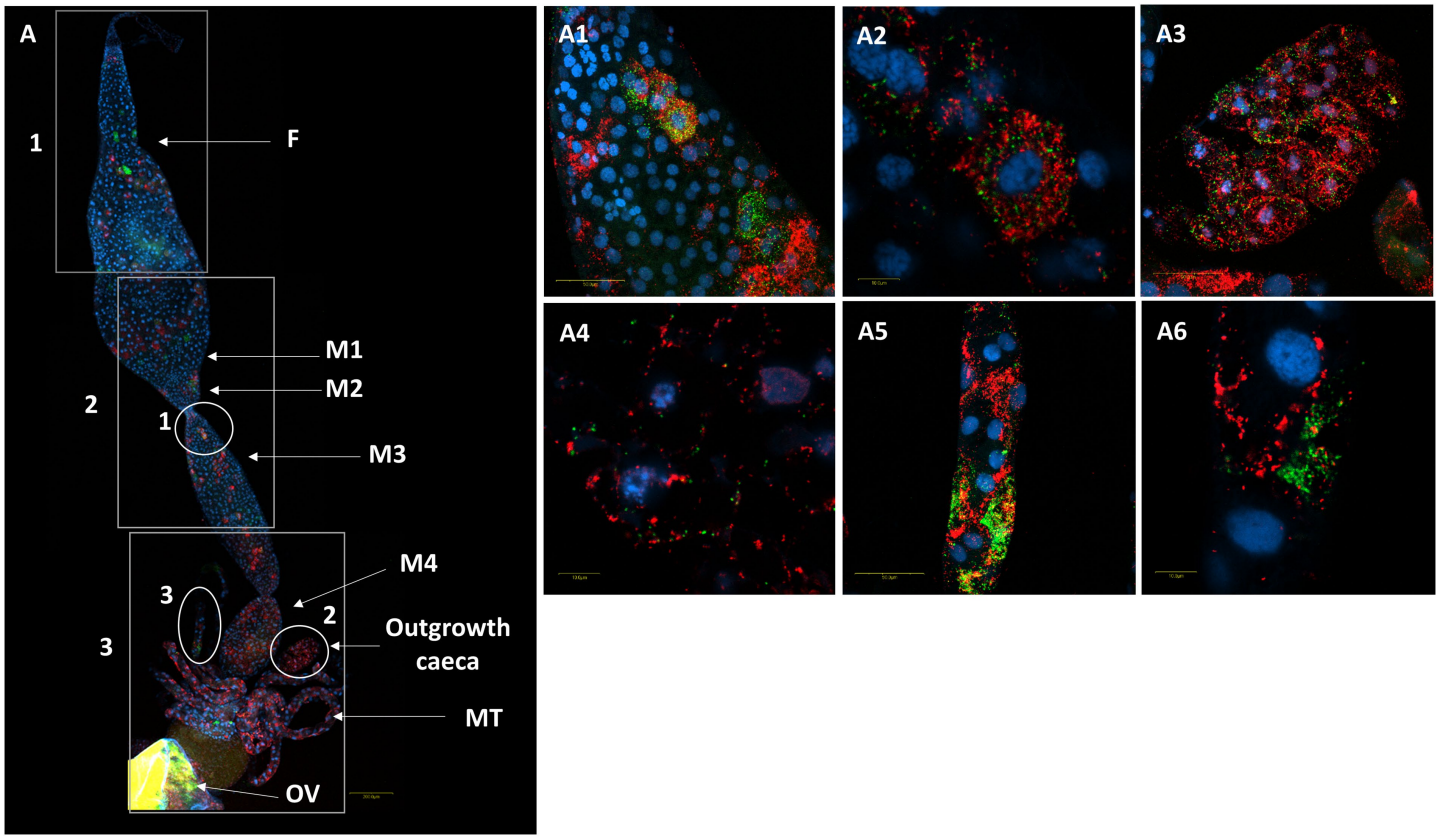
FISH of a female *M. melanotoma* digestive system (DNA in blue). **(A)** The whole digestive tract, *R. bellii* (red), *R. limoniae* (green), reconstructed from 3 images along the gut. Reconstruction: three frames of the same gut **(A)**; rectangle 1 picture number 1, rectangle 2 picture number 2, rectangle 3 picture number 3 with two outgrowth caeca from the gut tissue of the fourth midgut region. **(A1,A2)** Enlarged region of the midgut (circle 1 in A). **(A3,A4)** Enlarged region of the two outgrowth caeca (circle 2 in A). **(A5,A6)** Enlarged region of the Malpighian tubules (circle 3 in A). Images A number 1, A number 2, A number 3 and **(A1–A6)** represent serial Z section of 40 μm, 40 μm, 35 μm, 21 μm, 1 μm, 27 μm, 1 μm, 22 μm, and 1 μm respectively; F, foregut; M1, midgut first region; M2, midgut second region; M3, midgut third region; M4, midgut fourth region (with outgrowth caeca); MT, Malpighian tubules; OV, ovipositor.

A comparison of *M. pygmaeus* and *M. melanotoma* revealed the presence of large numbers of the two *Rickettsia* species throughout the digestive system in both females and males, with each *Macrolophus* species displaying a unique distribution pattern. In *M. pygmaeus, R. limoniae* was more broadly distributed along the host digestive system, while *R. bellii* was located primarily in the foregut and the midgut (Figure 6). In *M. melanotoma,* in contrast, *R. bellii* was more broadly distributed along the digestive system, while *R. limoniae* was clustered (Figures 7, 8). In both bug species, the two *Rickettsia* were usually isolated in separate host cells; they were, however, infrequently found sharing a common epithelial cell (Figure 8A2). FISH targeting bacterial *16S rRNA* visualized *R*. *limoniae* and *R. bellii* in the caeca-like organs, in both females and males (Figures 7A2, 8A3). Likewise, FISH analysis detected *R. bellii* and *R. limoniae* within the Malpighian tubules of females and males of both bug species (Figures 7A3, 8A5).

FISH analysis revealed the presence of *R. bellii* and *R. limoniae* in the anterior and posterior lobes of the salivary glands as well as the salivary duct in both species, with unique distribution patterns in each *Macrolophus* species. In *M. melanotoma*, *R. bellii* was more broadly distributed throughout the salivary gland, whereas *R. limoniae* appeared to be more clustered. In *M. pygmaeus*, this distribution pattern is reversed (Figure 4).

## 4. Discussion

In this study we detected, characterized and compared two symbiotic *Rickettsia* species, *R. limoniae* and *R. bellii*, inhabiting two *Macrolophus* species, *M. pygmaeus* and *M. melanotoma*. Phylogenetic analyses showed that *R. limoniae* from the two bugs studied most closely resemble each other, and share higher sequence similarity than to the symbiont sequenced from *M. pygmaeus* reported from the United Kingdom. Further, these *R. limoniae* sequences cluster closer to a bacterium sequenced from an Odonata species, than to those found in various other Hemiptera. A similar phylogenetic pattern was obtained for *R. bellii*, but based on the genes sequenced, it seems there is a clade that includes the symbiont found in Hemiptera, which all cluster together, with the exception of a strain from *Bemisia tabaci*. Altogether, the emerging pattern agrees with previous studies that suggest occasional horizontal transfer of *Rickettsia* (Nováková and Šmajs, 2019). Such a transfer may occur *via* feeding, either directly by the consumption of infected prey, or indirectly *via* feeding on host plants shared with other phytophagous insects. It is also possible that additional insect species feeding on the same host plants might take up microorganisms transferred by the bug to the plant. *Rickettsia* have been shown to be acquired from environmental sources by two species of *Spalangia* (Hymenoptera: Pteromalidae), *S. endius* and *S. cameroni* (Tzuri et al., 2021). Furthermore, Chrostek et al. (2017) reviewed the transmission of *Rickettsia, Wolbachia,* and *Cardinium* through plants by the leafhopper *Euscelidius variegatus*. These findings indicate that a co-evolutionary process between *Macrolophus* species and their symbiotic *Rickettsia* is unlikely.

In *M. melanotoma*, *R. bellii* is clustered with the bellii group based on the *CoxA* and *GltA* genes, but is more similar to the *R. limoniae* group according to the *16S rRNA* gene phylogeny. These results suggest a possible recombination between the two *Rickettsia* species (Jiggins, 2006). Recombination events are not rare in *Rickettsia* genomes (Wu et al., 2009; Merhej and Raoult, 2011), and play an important role in the evolution of these bacteria, notably by enabling them to adapt to new hosts (Thomas, 2016).

The two *Rickettsia* species were documented in all individuals of *M. pygmaeus* (Dally et al., 2020) and had a high rate of occurrence (84%) in screened *M. melanotoma*, adults. This is contrary to the findings of a previous study, in which only *R. limoniae,* was detected in *M. melanotoma* (Machtelinckx et al., 2012). This difference may stem from the different collection sites (Israel in the current study while Machtelinckx et al., 2012 collected in Greece and Italy), which may have various environmental conditions, selection pressures or infection histories. Moreover, this may be an indication of horizontal transfer of *Rickettsia* between the mirid species. Finding the two *Rickettsia* species in the salivary glands of their mirid hosts lends support to the possibility that the bacteria may be transferred to, and acquired from, their host’s food source. It is increasingly noted that bacteria can colonize the salivary glands of insects; this may have significant implications for plant–insect interactions, particularly for disease transmission by herbivorous insects (Kaiser et al., 2010; Body et al., 2013). Two well-documented examples are Citrus greening, the most destructive citrus disease in the world, caused by *Candidatus* Liberibacter asiaticus which resides in the salivary glands of the vector, the Asian citrus psyllid *Diaphorina citri* (Ammar et al., 2011), and Flavescence dorèe, a severe grapevine disease caused by *Candidatus* Phytoplasma vitis found in salivary glands of the leafhopper *Scaphoideus titanus* (Cicadellidae) (Marzorati et al., 2006). The endosymbiotic bacterium *Cardinium* was found to be injected into the plant by this leafhopper vector without any notable influence (Gonella et al., 2015). It can thus be seen that bacteria transmitted by herbivores to their host plants are not necessarily pathogenic. In some cases, bacterial symbionts have been shown to alter plant metabolic reconfiguration in a way that better meets insect nutritional needs. The endosymbiont *Wolbachia* has been shown to be transmitted by larvae of the leaf-mining moth *Phyllonorycter blancardella* to the leaves of apple seedlings (*Malus domestica)*, where the bacterium alters the phytohormonal profile of the leaves, creating an optimal microenvironment for its host (Kaiser et al., 2010; Body et al., 2013). Similar transmission was reported from another hemipteran, the sweet potato whitefly *Bemisia tabaci*, which transmits and acquires *Rickettsia* through the phloem of cotton plants (Caspi-Fluger et al., 2012).

The distribution of the two *Rickettsia* species in *M. melanotoma* ovarioles resembles their localization in *M. pygmaeus* (Machtelinckx et al., 2012; Dally et al., 2020). In both cases, the symbionts were found primarily in the germarium, strongly suggesting that the bacteria are transmitted vertically from the mother to her offspring via the egg (transovarial transmission), a common transmission pathway in many symbiont-host systems (Monti, 2017).

The two *Rickettsia* species displayed a unique distribution pattern in the two studied *Macrolophus* species. In *M. melanotoma, R. bellii* was distributed throughout the entire digestive tract, while *R. limoniae* appeared mainly in the foregut and the midgut. In *M. pygmaeus*, on the other hand, the distribution of *R. bellii* was more restricted than that of *R. limoniae*. Many phenotypic, genetic and physiological factors may lead to such variation in *Rickettsia* distribution in the gut. In addition, environmental factors such as seasonal conditions and host plants may also be involved. In the present study, for example, *M. pygmaeus* was reared in the laboratory on frozen *Ceratitis capitata* eggs and tomato seedlings under optimal temperature conditions, whereas *M*. *melanotoma* was collected in the spring from *Dittrichia viscosa* plants. Because species in the Miridae, especially those of the omnivorous Dicyphini tribe, are the best-known group of arthropods specialized for foraging, feeding, and oviposition on sticky plants (Wheeler and Krimmel, 2015), the difference in the food plant of the two studied hosts may influence *Rickettsia* involvement in the bugs’ nutritional ecology, and their distribution pattern might be influenced by the food source.

Mutualistic microbes often inhabit the caeca connected to the most anterior portion of the midgut (Hosokawa et al., 2016; Nardi et al., 2019), yet to the best of our knowledge such caeca structures have not been reported from any mirid species studied so far. Instead, our earlier work on *M. pygmaeus* (Dally et al., 2020), as well as the present study, revealed the presence of a paired organ at the posterior end of the midgut. Although this caecum-like organ appears to differ morphologically between the two studied *Macrolophus* species, as well as between males and females, it harbors both *Rickettsia* species in separate cells or, more rarely, together in the same cell. The function of these caeca-like organs is as yet unknown.

Obligate blood-feeders such as ticks, bed bugs, and tsetse flies have evolved to rely on microbial endosymbionts to supplement several B vitamins – such as biotin (B7), folate (B9), and riboflavin (B2) – that are deficient in blood. For example, the endosymbiont *Rickettsia buchneri* provides its tick hosts with biotin and folate, essential components for cell growth in all eukaryotic and prokaryotic organisms (Rio et al., 2016; Narasimhan et al., 2021).

In many hemipterans, symbiotic bacteria found on the midgut epithelia supply their hosts with essential nutrients such as amino acids and vitamins (Kikuchi et al., 2009), and recycle metabolic wastes of the host (Ohbayashi et al., 2019). The presence of *Rickettsia* in the digestive tract of both *M. pygmaeus* and *M. melanotoma,* as well as other omnivorous mirids, such as the midgut epithelial cells of *Stenotus binotatus* (Chang and Musgrave, 1970) and the lumen of the digestive tract of *Nesidiocoris tenuis* (Caspi-Fluger et al., 2014), may be indicative of the nutritional role filled by *Rickettsia* symbionts in this group. However, the omnivorous habit of these bugs ensures a balanced diet, so other phenotypes could not be ruled out. Examples could be found in Liu and Guo (2019), which reviewed the roles *Rickettsia* and *Wolbachia* play in protecting hosts from stresses caused by natural enemies, heat, and toxins. Moreover*, M. pygmaeus* became more sensitive to freezing conditions when all three symbionts, *Wolbachia, R. bellii* and *R. limoniae* were removed, but it is unclear the absence of which symbiont causes the negative effect (Maes et al., 2012).

Coinfections are well known for *Wolbachia* (Ant and Sinkins, 2018) but have been less commonly recorded for other symbionts. The presence of both *R. bellii* and *R. limoniae* in the two studied bug species is, however, in agreement with the widespread occurrence of co-infections in the Torix group of *Rickettsia* (Pilgrim et al., 2021). Co-infection with *Rickettsia* from the Torix group was also observed in the two damselflies *Coenagrion puella* and *Coenagrion pulchellum* (Thongprem et al., 2021).

In conclusion, the presence of two *Rickettsia* species with specific distribution patterns that differed between two *Macrolophus* host species was described. These symbiont species are phylogenetically similar and were found in the vast majority of the field-collected host bugs. As sequencing the genome of the two *Rickettsia* species could elucidate the role of such symbionts in the feeding habits of their hosts, it would be warranted to expand this research by combining lab experiments on feeding behavior with investigations into molecular genetics and bioinformatics.

## Data availability statement

The data presented in the study are deposited in the GenBank repository, accession numbers OQ374915-OQ374918, OQ374921-OQ374924, OQ398710, OQ398711, OQ271380-OQ271381 and OQ410975.

## Author contributions

MD: wrote the manuscript, performed, and designed the work. YZ: performed the phylogenetic analysis and wrote sections of the manuscript. EB: captured FISH images, and wrote the FISH method section. NM-D: contributed to the phylogenetic analysis section. MC and EZ-F: contributed to conception and design of the study. All authors contributed to manuscript revision, read, and approved the submitted version.

## Funding

This research was supported by the Israel Science Foundation (grant no. 397/21 to EZ-F).

## Conflict of interest

The authors declare that the research was conducted in the absence of any commercial or financial relationships that could be construed as a potential conflict of interest.

## Publisher’s note

All claims expressed in this article are solely those of the authors and do not necessarily represent those of their affiliated organizations, or those of the publisher, the editors and the reviewers. Any product that may be evaluated in this article, or claim that may be made by its manufacturer, is not guaranteed or endorsed by the publisher.
