# Supplementary material for: Rickettsia association with two Macrolophus (Heteroptera: Miridae) species: A comparative study of phylogenies and within-host localization patterns
Source: Front Microbiol. 2023 Feb 23;13:1107153. doi: 10.3389/fmicb.2022.1107153 (PMC9998071; doi:10.3389/fmicb.2022.1107153)
Supplement: Supplementary file 4 [file Data_Sheet_1.docx]

**Table:** Collection sites of *Macrolophus melanotoma* female from *Dittrichia viscosa* plants in Israel (2021).

| Locality | Habitat | Coordinates | Collection date | N |
| --- | --- | --- | --- | --- |
| Galilee | Roadside | 32.84796° N, 35.24686°E | 31.03 | 20 |
| Golan height | Scrubland | 32.88169° N, 35.63222°E | 15.03 | 12 |
| Jordan river | Riverbank | 32.90286° N, 35.54000°E | 5.04 | 25 |
| Galilee | Home garden | 32.90412° N, 35.55065°E | 15.03 | 13 |
| Shfela | Roadside | 31.83187° N, 34.88239°E | 25.03 | 12 |
