# Supplementary figures and images for: Rickettsia association with two Macrolophus (Heteroptera: Miridae) species: A comparative study of phylogenies and within-host localization patterns
Source: Front Microbiol. 2023 Feb 23;13:1107153. doi: 10.3389/fmicb.2022.1107153 (PMC9998071; doi:10.3389/fmicb.2022.1107153)

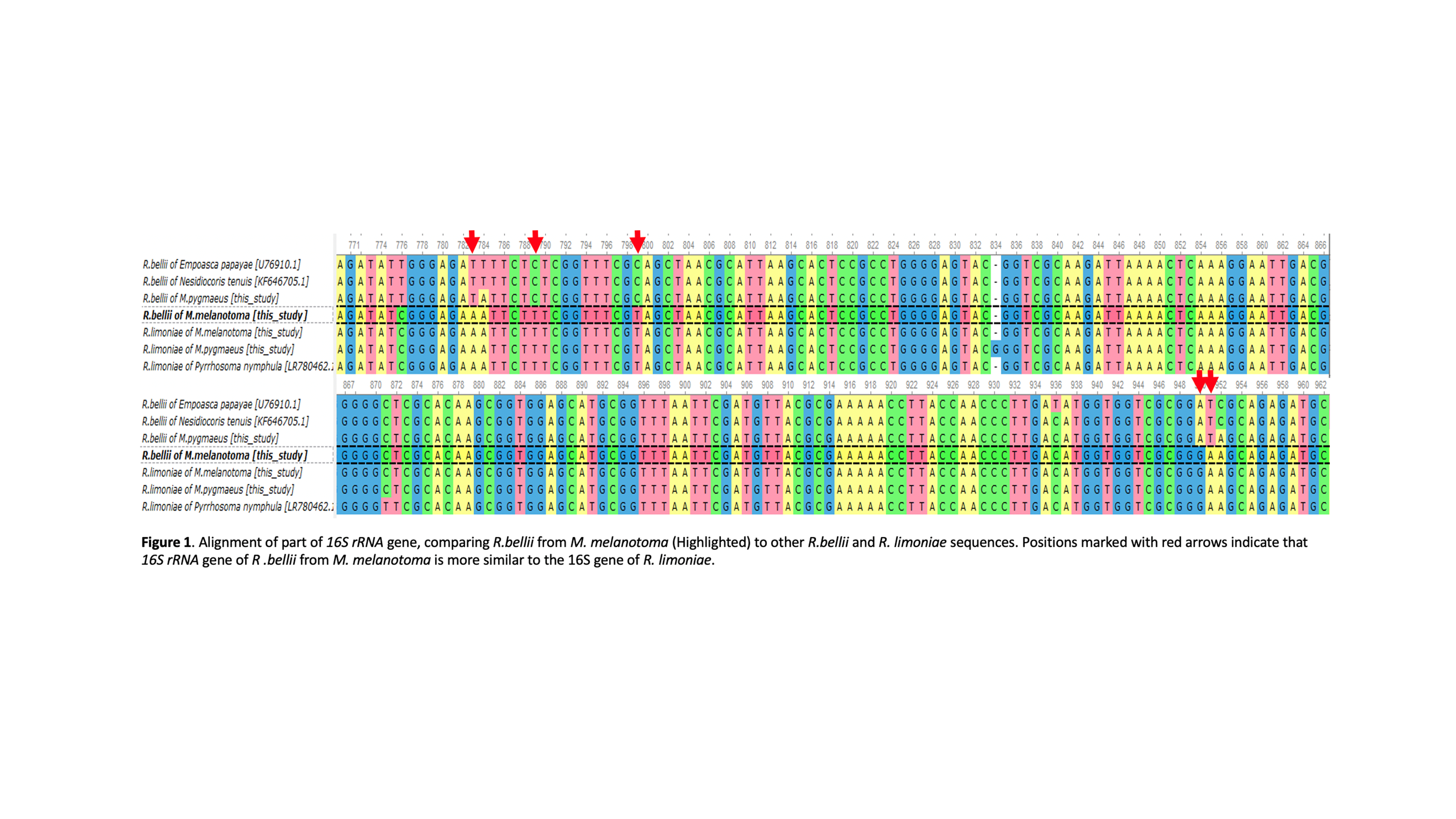

Supplement: Supplementary file 5 [file Image_1.TIF]
